# Complete Genome Sequence of *Campylobacter gracilis* ATCC 33236^T^

**DOI:** 10.1128/genomeA.01087-15

**Published:** 2015-09-17

**Authors:** William G. Miller, Emma Yee

**Affiliations:** Produce Safety and Microbiology Research Unit, Agricultural Research Service, U.S. Department of Agriculture, Albany, California, USA

## Abstract

The human oral pathogen *Campylobacter gracilis* has been isolated from periodontal and endodontal infections, and also from nonoral head, neck, or lung infections. This study describes the whole-genome sequence of the human periodontal isolate ATCC 33236^T^ (=FDC 1084), which is the first closed genome for *C. gracilis*.

## GENOME ANNOUNCEMENT

*Campylobacter gracilis* (formerly *Bacteroides gracilis* [1]) is generally associated with the human oral environment and has been isolated from both periodontal and endodontal infections (2–4); however, this organism has also been isolated from cases of nonoral deep tissue infection (5). The *C. gracilis* type strain ATCC 33236 was isolated from the gingival sulcus of a human with periodontitis (1). Genome data for this strain (deposited as RM3268) is currently present within GenBank as a draft scaffold of 33 contigs. In this study, we report the first closed whole-genome sequence of strain ATCC 33236^T^.

The Roche GS-FLX and Illumina HiSeq sequencing platforms were used to complete the *C. gracilis* strain ATCC 33236^T^ genome. A total of 237,579 shotgun and paired-end Roche 454 reads (55× coverage) were assembled, using the Newbler assembler version 2.6, into one scaffold of 18 contigs (18 scaffold contigs interspersed with 17 unique contigs and 11 repeat contigs). Sanger DNA sequencing of contig-bridging amplicons was used to close the scaffold into a single contig. All 454 base calls were validated using 14,326,852 HiSeq reads (SeqWright, Houston, TX, USA), adding a coverage of 634× (689× final coverage). An optical restriction map (OpGen, Gaithersburg, MD, USA) with restriction enzyme BamHI was used for assembly validation.

*C. gracilis* strain ATCC 33236^T^ has a circular genome of 2,281,652 bp with an average GC content of 46.55%. Protein-, rRNA-, and tRNA-encoding genes were identified as described previously (6). The genome encodes 2,103 putative protein-coding genes, 34 pseudogenes, and 3 rRNA operons. The genome contains two CRISPR/Cas loci, two integrated phages, and no contingency genes. Additionally, no plasmids were identified in this strain.

A unique feature of the *C. gracilis* type strain is its predicted respiratory profile. The high O_2_ affinity CcoNOPQ cytochrome *c* oxidase is absent. Absent also are the NrfAH nitrite reductase and the FdhABC formate dehydrogenase. However, this strain does contain two formate dehydrogenase H genes linked to the *nap* nitrate reductase and *hyf* hydrogenase-4 loci, and encodes an alternate formate dehydrogenase N. Strain ATCC 33236^T^ is also predicted to encode the HydABC NiFe hydrogenase, the NorZ nitric oxide reductase, the NosZ nitrous oxide reductase, a carbon monoxide dehydrogenase, a thiosulfate/polysulfide reductase, a tartrate dehydratase, a putative tartrate dehydratase/fumarate hydratase, as well as other uncharacterized respiratory enzymes.

*C. gracilis* strain ATCC 33236^T^ does not contain the *acn*, *gltA*, *icd*, or *sucCD* TCA cycle genes; these genes are similarly absent from members of the *C. lari* clade (6). Also, this strain does not contain genes encoding flagellar, flagellar modification, or chemotaxis proteins.

The *C. gracilis* type strain is however predicted to encode a number of putative genes associated with pathogenicity. These include hemagglutinins, toxins, immunity proteins, and other predicted virulence factors. This strain also contains the zonula occludens toxin (zot) genomic island (7). Although *C. gracilis* is generally not considered an important pathogen, the presence of these virulence determinants suggests that this species warrants further investigation.

### Nucleotide sequence accession number.

The complete genome sequence of *C. gracilis* strain ATCC 33236^T^ has been deposited in GenBank under the accession number CP012196.
